# Neonatal Presentation of Ornithine Transcarbamylase Deficiency Associated With a Hypomorphic OTC Variant (p.Leu301Phe) Previously Reported in Later-Onset Disease

**DOI:** 10.7759/cureus.65956

**Published:** 2024-08-01

**Authors:** Sharon Anderson, Molly Ciarlariello, Christina Botti, Milen Velinov

**Affiliations:** 1 Medical Genetics, Rutgers Health, Robert Wood Johnson Medical School, New Brunswick, USA; 2 Division of Advanced Nursing Practice, Rutgers School of Nursing, Newark, USA; 3 Obstetrics, Gynecology, and Reproductive Sciences, Rutgers Health, Robert Wood Johnson Medical School, New Brunswick, USA

**Keywords:** ornithine transcarbamylase deficiency, ornithine carbamoyltransferase deficiency, x-linked hyperammonemia, urea cycle disorder, otc deficiency

## Abstract

Ornithine transcarbamylase deficiency (OTCD) is the most common subtype of urea cycle disorders. Caused by mutations in the X-linked gene *OTC*,it often leads to hyperammonemia which can result in neurotoxicity, coma, and death. We describe the clinical course of a male newborn known to carry a hypomorphic variant (p.Leu301Phe) in *OTC *previously reported in cases with later-onset OTCD. Despite being clinically asymptomatic, our affected patient presented with hyperammonemia in the neonatal period. Oral feedings were temporarily discontinued, and low protein medical formula and ammonia scavenger medications were initiated to normalize ammonia levels. This case supports the pathogenicity of the reported *OTC* gene variant and early presentation that necessitates disease-specific management. Our report will help provide guidance surrounding the most appropriate management of future patients with this variant as they will likely require management in the newborn period.

## Introduction

Ornithine transcarbamylase deficiency (OTCD) (MIM: 311250) is an X-linked dominant urea cycle disorder with a prevalence of 1:14,000-77,000 live births [1]. Variants in the *OTC* gene located on chromosome Xp11.4 block the OTC enzyme from completing a sequence of reactions in the liver to convert ammonia into urea and to facilitate the excretion of ammonia by the kidneys [2]. This deficiency leads to the classic symptom triad of hyperammonemia, encephalopathy, and respiratory alkalosis [3].

There are more than 500 known *OTC* gene variants [4]. Although genotype-phenotype correlations exist, individuals with OTCD have a variable phenotype with presentation across the lifespan [3,4]. Hemizygous males may present with severe, neonatal-onset, hyperammonemia crisis with blood ammonia levels over 340 µg/dL (200 µmol/L) that can cause neurological damage and poor neurodevelopmental outcomes ranging from developmental delay to intellectual disability and executive function deficits [3,5]. Of note, if a fetus/newborn is known to carry a neonatal-onset variant, timely treatment and early liver transplant improve quality of life and may help achieve normal outcomes [3,5]. Alternatively, males who carry a variant resulting in partial OTC enzyme activity may present later in life with hyperammonemia progressing to encephalopathy and coma [3]. Due to X-linked inactivation, heterozygous females with pathogenic variants may develop an array of manifestations ranging from asymptomatic to severe hyperammonemia attacks with onset from infancy to adulthood [1,3,6].

In the non-acute setting, treatment for OTCD involves dietary protein restriction, essential amino acid metabolic formula, low protein medical foods, supplemental amino acids, nitrogen scavenger medications, or orthoptic liver transplantation which is curative. Although not yet widely available, gene replacement and editing therapy modalities show great promise [6,7].

## Case presentation

A full-term male infant was born to a 30-year-old G2 P 1001 -> 2 asymptomatic OTCD carrier female. His mother was a known carrier for a likely pathogenic variant (c.903A>T, p.Leu301Phe) identified through Inheritest® 500 panel during her first pregnancy. At the time, she underwent genetic counseling, and a detailed family history revealed no evidence of OTCD. Familial cascade testing was initiated, and while the mutation was found to be maternally inherited, no males with the variant were found in the maternal family out to third-degree relatives. However, there were few males in the extended family. The couple was offered family planning interventions such as in-vitro fertilization with preimplantation genetic testing for monogenetic/single-gene diseases but declined.

Amniocentesis during the first pregnancy revealed a non-carrier female fetus, and, as such, neonatal monitoring was not indicated. Surveillance for maternal hyperammonemia was initiated based on evidence that heterozygous females may develop hyperammonemia due to increased metabolic demands of pregnancy, parturition, and early postpartum period [4,8]. The same management plan was enacted during the second pregnancy. Again, she experienced no hyperammonemia.

During this pregnancy, maternal prenatal labs were unremarkable and ammonia levels were monitored and remained within normal limits. Noninvasive prenatal testing revealed a male karyotype (46,XY), and amniocentesis confirmed the male fetus carried the known familial variant c.903A>T (p. Leu301Phe). Fetal karyotype and microarray were normal. Delivery was induced at 39 weeks. He was born by spontaneous vaginal delivery and Apgar scores were 9 and 9 at one and five minutes, respectively, requiring only tactile stimulation at birth. His birth weight was 3,105 g (30th percentile).

Upon admission to the neonatal intensive care unit, an umbilical venous line was placed. Admission hemoglobin and hematocrit levels were 13 g/dL and 37.4%, respectively. The initial ammonia level was 117 µg/dL (68.7 µmol/L), an acceptable range for a newborn. The first citrulline level was normal (7.9 µmol/L, LabCorp reference range for age: 6.6-25.8 µmol/L), and glutamine levels were low (323.2 µmol/L, LabCorp reference range for age: 333.0-810.2 µmol/L). The orotic acid level was 3.0 mmol/mol Cr (LabCorp reference range: 1.4-5.3 mmol/mol Cr). Postnatal labs showed ABO incompatibility and positive Coombs (O-/B-/C+). He was treated with phototherapy from days three to four. Phototherapy was initiated for a total bilirubin of 8.7 mg/dL on day three with a peak of 10.6 mg/dL on day seven.

Initially, the newborn was provided breast milk (including donor breast milk when the mother’s milk was unavailable). By day four, ammonia levels started to rise and the citrulline level decreased to 3.7 µmol/L (Lab Corp reference range for age: 6.6-25.8 µmol/L). Enteral feedings were stopped and intravenous fluids of 10% dextrose at 1.5 maintenance and intravenous arginine were initiated. Once the ammonia levels returned to a more acceptable range, UCD Anamix® Infant formula (Nutricia Metabolics North America) was introduced, followed by glycerol phenylbutyrate. By day six, ammonia levels stabilized. Fluids were decreased to maintenance, a small amount of breast/formula was introduced, and the ammonia level frequency was tapered. By day seven, the baby had returned to his birth weight, and intravenous arginine was switched to oral. On day nine, a breast milk challenge (10 mL of breast milk with each feeding) was attempted but was unsuccessful, requiring a return to metabolic formula due to rising ammonia levels.

Over the next 10 days, a balance of metabolic formula, breast milk, glycerol phenylbutyrate, and arginine was successful in stabilizing ammonia levels. During the hospital course, the patient’s highest ammonia level was 228 µg/dL (133.88 µmol/L). For several days before discharge, ammonia levels remained between 79 and 113 µg/dL (46.39-66.35 µmol/L). Over the treatment course, glycerol phenylbutyrate adjustments were made and oral arginine was switched to citrulline (day 14).

The patient was discharged on day 19. The treatment plan included ammonia and plasma amino acid levels outpatient (the evening of discharge or next day), UCD Amamix formula (essential amino acid formula developed for the treatment of urea cycle disorders) ad lib with breast milk feedings 10 mL every 12 hours, glycerol phenylbutyrate 0.8 mL PO TID, and citrulline powder 150 mg every eight hours. Follow-up with genetics was scheduled to facilitate ongoing assessment of ammonia levels and optimization of intact protein feeding advancements.

There have been two genetic visits since discharge. The ammonia levels have remained stable, and, as such, intact protein feedings of breast milk feedings (including opportunities for mom to nurse) have been increased. At the writing of this manuscript, the patient is two months old. He is receiving 50% metabolic formula, 50% intact protein from breast milk, and oral citrulline and glycerol phenylbutyrate with stable ammonia levels. During the entire inpatient course, the patient remained clinically asymptomatic.

## Discussion

A search of the literature in the prenatal setting identified three case reports of male individuals with the c.903A>T (p.Leu301Phe) variant. The first was reported by Capistrano-Estrada et al. (1994) and cited again in a case report by Climent and Rubio (2002). This male first presented at age 12.5 years with acute hyperammonemia with altered mental status, including combative behaviors and icteric sclera. Plasma ammonia level was 282 µmol/L (480.25 µg/dL) and percutaneous liver biopsy showed 3% residual OTC activity [9,10].

The second individual was reported in 2012. This 35-year-old male was admitted with vomiting, confusion, and agitation following intense physical exertion (cycling). The history was non-contributory, but the patient reported a brother who died at age five from a neurologic and hepatic disease. Upon admission, there was rhabdomyolysis, and his ammonia level was 256 µmol/L (436 µg/dL; reference range: 16-50 µmol/L). Genetic testing confirmed the diagnosis of OTCD caused by the same variant [11].

The third and most recent case report of a patient with this variant was published by Lung and colleagues (2022). This 50-year-old male presented with acute encephalopathy (altered mentation and dysarthria), nausea, insomnia, and headache two days post-surgical correction of strabismus. Routine treatment including laboratory testing and brain CT and MRI scans were completed and unremarkable. Intravenous antibiotics and antivirals were started. With continued agitation, an ammonia level was checked and found to be 260 µg/dL (152.67 µmol/L). He developed respiratory alkalosis and his mental status deteriorated requiring intubation for airway protection within 24 hours of admission and emergent hemodialysis. His highest ammonia level was 608 µg/dL (354 µmol/L). Probing the past medical history, the patient reported a Reye-like syndrome episode at age eight (encephalopathy after a viral illness) and acute encephalopathy (transient global aphasia) at age 32. His acute clinical presentation was attributed to preoperative fasting and catabolism followed by high protein intake [12]. Table 1 provides a comparison of the available clinical details of all four male patients with c.903A>T, p. Leu301Phe *OTC* variants.

**Table 1:** Overview of male patients with c.903A>T, p.Leu301Phe OTC variant. OTC = ornithine transcarbamylase

	Capistrano-Estrada et al. (1994) [9]	Morel et al. (2012) [11]	Lung et al. (2022) [12]	Anderson et al. (2024)
Age at onset (years)	12.5	35	50	0
Presenting symptoms	Altered mental status, combative behaviors, icteric sclera	Vomiting, confusion, agitation	Altered mental status, dysarthria, nausea, insomnia, headache two days post-surgical strabismus correction	Clinically asymptomatic
Triggers	Two-day prodrome of vomiting and lethargy	Intense physical exertion (cycling)	Preoperative fasting followed by high protein intake	-
Ammonia level	282 µmol/L (440.25 µg/dL)	256 µmol/L (436 µg/dL)	152.67 µmol/L (260 µg/dL)	133.88 µmol/L (228 µg/dL)
Confirmed diagnosis	Percutaneous liver biopsy showed 3% residual OTC enzyme activity	*OTC* gene sequencing	*OTC* gene sequencing and deletion/ duplication	*OTC* familial variant testing (amniocentesis)
Comments	-	Confirmed the same variant as his brother who succumbed to OTC deficiency at age five	Past medical history revealed Reye-like syndrome at age eight and acute encephalopathy at age 32	-

## Conclusions

To our knowledge, this is the fourth male patient with the c.903A>T (p.Leu301Phe) *OTC* gene variant to be reported in the literature and the first to be diagnosed in a newborn. Given the available evidence, in the prenatal setting, we recommended a conservative approach including close monitoring of our patient after delivery. Although newborn screening for OTCD is included in several US state newborn screening programs, screening methods and sensitivity and specificity vary by state and cannot reliably identify all cases. Furthermore, male infants with neonatal-onset variants may be acutely ill and encephalopathic before the newborn screen result is reported. As such, this case highlights the benefits of prenatal diagnosis and approaching all male newborns with partial *OTC* gene variants with caution and maintaining a high index of suspicion. Treating each at-risk individual as unique (even when disease presentation/symptoms are unanticipated) is prudent. While not anticipated, this clinically asymptomatic newborn developed hyperammonemia and required treatment. His mother’s expanded carrier screening, prenatal diagnosis, and subsequent pregnancy and postnatal plans for her and her son allowed us to identify rising ammonia levels and initiate timely treatment that mitigated the risk for metabolic crisis and/or neurologic sequelae for this newborn.
